# The health impact of human papillomavirus vaccination in the situation of primary human papillomavirus screening: A mathematical modeling study

**DOI:** 10.1371/journal.pone.0202924

**Published:** 2018-09-04

**Authors:** Suzette M. Matthijsse, Steffie K. Naber, Jan A. C. Hontelez, Roel Bakker, Marjolein van Ballegooijen, Iris Lansdorp-Vogelaar, Inge M. C. M. de Kok, Harry J. de Koning, Joost van Rosmalen, Sake J. de Vlas

**Affiliations:** 1 Department of Public Health, Erasmus MC, University Medical Center Rotterdam, Rotterdam, the Netherlands; 2 Heidelberg Institute of Public Health, Faculty of Medicine, Heidelberg University, Heidelberg, Germany; 3 Department of Biostatistics, Erasmus MC, University Medical Center Rotterdam, Rotterdam, the Netherlands; Rudjer Boskovic Institute, CROATIA

## Abstract

**Background:**

Human papillomavirus (HPV) vaccination and the implementation of primary HPV screening in the Netherlands will lead to a lower cervical disease burden. For evaluation and further improvement of prevention, it is important to estimate the magnitude and timing of health benefits of current and alternative vaccination strategies such as vaccination of boys or adults.

**Methods and findings:**

We evaluated the impact of the current girls-only vaccination program and alternative strategies on cervical disease burden among the first four vaccinated five-year birth cohorts, given the context of primary HPV screening. We integrated the existing microsimulation models STDSIM (HPV transmission model) and MISCAN-Cervix (cervical cancer screening model). Alternative vaccination strategies include: improved vaccination uptake, including routine boys vaccination, and offering adult vaccination at sexual health clinics. Our models show that the current vaccination program is estimated to reduce cervical cancers and cancer deaths by about 35% compared to primary HPV screening in the absence of vaccination. The number needed to vaccinate (NNV) to gain 1 life year is 45. The most efficient alternative vaccination strategies are: 1) improving coverage of girls to 80% (NNV = 42); and 2) routine vaccination for girls and boys at 80% coverage (incremental NNV = 155), with cervical cancer mortality reductions estimated at 50% and 60% respectively.

**Conclusions:**

While the current program already substantially reduces cervical cancer incidence and mortality, prevention can be further improved by increasing vaccination uptake and extending vaccination to boys. As not all cervical cancer deaths will be prevented, screening participation should still be encouraged.

## Introduction

Cervical cancer is the fourth most common cancer among women worldwide [1], with the human papillomavirus (HPV) as its necessary cause. Over the past years, several countries have implemented vaccination against the most oncogenic types HPV-16 and HPV-18. While clinical trials indicated that the currently licensed bivalent, quadrivalent, and nonavalent vaccines are highly effective against HPV-16 and HPV-18 infection [2–4], coverage levels in most countries remain rather disappointingly low [5]. However, by reducing HPV prevalence in the population, unvaccinated women are (indirectly) protected as well (i.e. herd immunity) [6, 7]. We recently estimated that HPV-16 and HPV-18 incidence in the Netherlands will substantially decline by about 60% under continuation of the current girls-only vaccination program [7]. Alternative vaccination strategies, such as efforts to increase vaccination coverage among girls, the inclusion of boys, or vaccinating men at sexual health clinics, have also been explored and in some countries even implemented [8, 9]. Finally, the cervical cancer screening program in the Netherlands was renewed in 2017 by offering primary HPV screening instead of cytology to women starting at age 30, and by offering fewer lifetime screens [10]. Given the context of new screening strategies, it is important to evaluate the health benefits of current and alternative HPV vaccination strategies in order to support decision making on improving HPV vaccination uptake and targeting.

Mathematical modeling has been used to estimate HPV incidences and the associated cervical disease burden following vaccination, as well as to determine the most cost-effective cervical cancer screening strategy in the post-vaccination era [11–23]. However, in order to accurately estimate the impact of vaccination, including herd-immunity and the complexity of cervical cancer disease development, a comprehensive modeling approach that combines a sexual network model for accurately predicting vaccination effects on HPV transmission and a detailed model of cervical disease natural history and screening is crucial. Most models used did not simulate HPV transmission through a sexual network [11–20], and therefore cannot properly capture herd immunity effects, or did not specifically capture the complexity of cervical cancer disease progression and screening [9].

We developed an HPV transmission and cervical cancer screening modeling framework by integrating two established and comprehensive microsimulation models of HPV transmission (STDSIM) and cervical cancer development (MISCAN-Cervix). With this new modeling framework, we estimated the cervical disease burden for the first cohorts who have received HPV vaccination under the new cervical cancer screening program, while taking both the direct and indirect effects of HPV vaccination into account. In addition, we determined the change in cervical cancer burden under various foreseeable extensions of the current girls-only vaccination program.

## Methods

### STDSIM model

STDSIM is an established stochastic microsimulation model of the spread and control of sexually transmitted infections (STIs) [24–26]. The model simulates the life course of individuals in a dynamic heterosexual network, in which STIs such as HPV can spread. Each individual has its own characteristics that are either constant (e.g. date of birth and sex) or subject to change (e.g., number of sexual partners, infection status). Events are determined by probability distributions, and can lead to new events (e.g. birth leads to a future event of becoming sexually active) or a cancellation of future events (e.g. death cancels all scheduled events concerning sexual activity or STI transmission). More detailed information on the model can be found in Hontelez *et al*.[25].

We have previously quantified STDSIM to the Netherlands to model the spread of HPV-16 and HPV-18. Briefly, we reproduced the Dutch population and its sexual network, based on demographic and sexual data. As an independent validation of the underlying sexual network, we simulated the transmission of chlamydia to validate the level of sexual risk behavior. The model was able to closely reproduce the observed chlamydia prevalence levels, reassuring us that the simulated sexual network is representative for the Dutch situation. We then introduced HPV-16 and HPV-18 in the population to estimate the transmission probabilities and acquired immunity dynamics necessary to reproduce the age-specific patterns in HPV-16 and HPV-18 prevalence. All details on the model structure, the parameter quantification, and model validation and underlying data for the Dutch setting, are described in detail by Matthijsse *et al*. [26]

### MISCAN-Cervix model

MISCAN-Cervix simulates the individual life histories of a population of women, based on Dutch demographic and hysterectomy data. Women can acquire a high-risk HPV infection that either clears naturally or leads to the development of pre-invasive cervical lesions. These lesions regress or develop into invasive cervical cancer. Death can follow from cervical cancer or from other causes. Multiple infections can occur at the same time, which are independent of each other. Interventions such as hysterectomy and screening can affect these life histories. Pre-invasive stages and FIGO (International Federation of Gynecology and Obstetrics) 1A cases can only be detected by screening, since they are assumed to be asymptomatic, whereas FIGO 1B or worse can also be clinically diagnosed [27].

The model divides cervical disease into seven sequential stages: high-risk HPV infection, three pre-invasive stages (cervical intraepithelial neoplasia (CIN) grade 1, 2, and 3), and three invasive stages (FIGO stages 1A, 1B, and 2 or worse; S2 Fig). Age-specific onset parameters set the probability of women to acquire an HPV infection and develop lesions or cancer. In the model, most HPV infections are transient. Lesions in pre-invasive stages can also regress. While CIN 1 and CIN 2 can develop without an HPV infection (in which case they will always regress in our model), CIN 3 and cervical cancer can only develop in the presence of a high-risk HPV infection. More information can be found in S1 Text, De Kok *et al*. [28], Van Rosmalen *et al*. [29], and Naber *et al*. [27].

### Integrated modeling framework

Using STDSIM, we estimated the relative reduction in incidence of HPV-16 and HPV-18 for a specific vaccination strategy over time and for different cohorts, compared to no vaccination. To determine the effect of vaccination on cervical precancerous lesions and cancer, we subsequently used these estimates from the STDSIM model as input for the MISCAN-Cervix model.

In previous studies with the MISCAN-Cervix model, an aggregate probability to acquire an HPV infection and develop a lesion or cancer was defined for all high-risk HPV types [25, 27, 28]. For the purpose of this study, we divided them into separate probabilities for HPV-16, HPV-18, and the other high-risk types to reproduce the observed attributable proportions of HPV-16 and HPV-18 in high-risk HPV prevalence, lesions and cervical cancer, while we maintaining the original aggregate values of these probabilities. The observed attributable proportions of HPV-16 in CIN 1, CIN 2, CIN 3, and invasive cervical cancer are 15.4%, 37.6%, 47.2%, and 62.5%, respectively, after correcting for co-infections with multiple types (S8 Table) [27, 30]; and 7.8%, 7.4%, 4.7%, 17.2% for HPV 18, respectively. Of the transient high-risk HPV infections, 25.4% is attributable to HPV-16 and 8.2% to HPV-18 [31]. In an unvaccinated population, the estimated proportion of HPV-16 and HPV-18 in transient high-risk HPV infections, precancerous lesions, and invasive cervical cancers in MISCAN-Cervix correspond well with the observed proportions, except for an underestimation of CIN 2 attributable to HPV-16 and a slight overestimation in CIN 1 and CIN 3 attributable to HPV-18 (S8 Table).

With the integrated modeling framework we simulated a population of 10 million women divided over four birth cohorts in MISCAN-Cervix with the following birth years: 1993–1997, 1998–2002, 2003–2007, and 2008–2012. These birth cohorts were chosen because they are the first vaccinated cohorts in the Netherlands. The trends in relative reductions in age-specific incidence of HPV-16 and HPV-18 from STDSIM per birth cohort, which incorporates both the direct and indirect effects of vaccination, were then applied to the age-specific onset parameters in the MISCAN-Cervix model (S4 Fig).

### Vaccination strategies

We used largely the same assumptions to model the impact of HPV vaccination as in our previous studies [7, 9]. Briefly, we modeled the current vaccination strategy and observed vaccination uptake in the Netherlands: i.e. a catch-up campaign for girls aged 13–16 years in 2009 (coverage of 50%), and annual vaccination of girls aged 12 years (coverage of 60%). Vaccine efficacy is set at 94.7% for HPV-16 and at 92.3% for HPV-18 [3, 32]. We assumed vaccine efficacy to be independent of HPV status at the time of vaccination, as vaccination still has a substantial protective effect in women previously exposed to HPV-16 and HPV-18 [33, 34]. Infection clearance is not accelerated by the vaccine in our model.

The alternative vaccination strategies represent foreseeable changes of the current girls-only program in various ways from 2017 onwards. First, we increased routine vaccination coverage among girls up to 80% and 100%, as the familiarity of the vaccine has increased and more girls might be inclined to get vaccinated in the future. Second, we included routine vaccination for boys, assuming the same target age groups and uptake as for girls (i.e. 60%, 80%, and 100% coverage). However, as bivalent vaccine efficacy estimates for boys are unavailable, efficacy for boys was assumed to be equal to the quadrivalent vaccine efficacy for boys (78.7% for HPV-16; 96.0% for HPV-18) [35]. Finally, we included adult vaccination at sexual health clinics for women and for both men and women aged 15–29 years. For the adult vaccination strategies, we assumed an efficacy of 77.4% against both HPV-types for women older than 24 years [34]. For men older than 24 years, vaccine efficacy was assumed to be 64.5% for HPV-16 and 80.6% for HPV-18 [9, 34]. In the last strategy, where the vaccine is offered to both sexes during STI consultations, routine vaccination for boys is included as well.

### Cervical cancer screening

We subsequently simulated the new cervical cancer screening program, in which primary HPV screening with reflex cytology and cytology triage after 6 months will be offered to women aged 30, 35, 40, 50, and 60 years. Additional HPV testing will be offered at age 45 and 55 for women who either have a positive HPV test or do not attend screening at ages 40 and 50, respectively. Women who attended screening at age 60 and tested positive for HPV will also be invited at age 65. We assumed that 10% of the population never attends screening and has a higher background risk than the 90% potential attenders [29]. Attendance among the potentially attending women for primary testing and compliance with colposcopy referrals and triage testing is based on the current screening program (see also S9 Table), and is independent of vaccination status in our model [36]. In the new screening program, a self-sampling kit is offered to women who do not attend screening at the general practitioner. Gök *et al*. estimated that the effect of mailing self-sampling kits to all non-attendees of the Dutch cervical cancer screening program would generate an extra 5.2% attendance [37]. Since the self-sampling kit will be offered using opt-in (instead of opt-out as in the PROHTECT trial), we assumed that 3% of the non-attenders would opt-in for the self-sampling kit [38].

We used the same specificity and sensitivity of cytology for detecting precancerous lesions and invasive cancer for testing at least atypical squamous cells of undetermined significance (ASC-US) as in our previous study, i.e. a sensitivity of 40% for CIN 1, 50% or CIN 2, and 75% for CIN 3 and cervical cancer, and a specificity of 98% [27, 39]. For the HPV test, we assumed a 94% sensitivity for detecting high-risk HPV infections, regardless of whether a CIN lesion or cancer was also present (although a woman with cancer is always infected with HPV) [26, 29]. In our model, CIN treatment leads to full recovery. Subsequently, women can acquire new HPV infections and develop CIN lesions and/or invasive cancer. For invasive cancer, survival probabilities depend on the woman’s age and cancer stage (FIGO 1B or FIGO 2+) at diagnosis, based on data from the Dutch Cancer Registry [40]. After receiving treatment for cancer, women are no longer at risk for HPV or cervical disease. The survival probabilities by age and stage can be found in Naber *et al*.[27].

### Model outcomes

STDSIM provides us with the estimated age-specific HPV-16 and HPV-18 incidence and vaccinated individuals over time. MISCAN-Cervix determines the most severe state of each woman (i.e. from least to most severe: normal, HPV infected, CIN 1, CIN 2, CIN 3, FIGO 1A, FIGO 1B, and FIGO 2+) and the number of life years spent in each state (see also S1 Text). For the current study, the main outcomes are the relative reductions in HPV-16 and HPV-18 incidence over time, and the numbers of diagnosed CIN 1, CIN 2, CIN 3 and cervical cancer, and cancer-related deaths. We also estimated the (incremental) number needed to vaccinate (NNV) to gain one life year, in order to determine the most efficient vaccination strategies.

### Sensitivity analyses

Huijsmans *et al*. [41] recently showed that HPV prevalence in screening eligible women in the Netherlands could be twice as high compared to the earlier data our model fit was based upon [42]. Therefore, we re-analyzed the impact of the current vaccination program in the models with doubled HPV prevalence prior to the implementation of vaccination in the sensitivity analyses. The overall prevalence among women was doubled by increasing sexual risk behavior in STDSIM as well as by doubling the onsets of transient infections in MISCAN. Also, we varied the attendance of the screening program by assuming a 20% higher and lower attendance (S9 Table), as we cannot be sure that screening attendance of the new program will be the same as of the current program.

## Results

**Fig 1:**
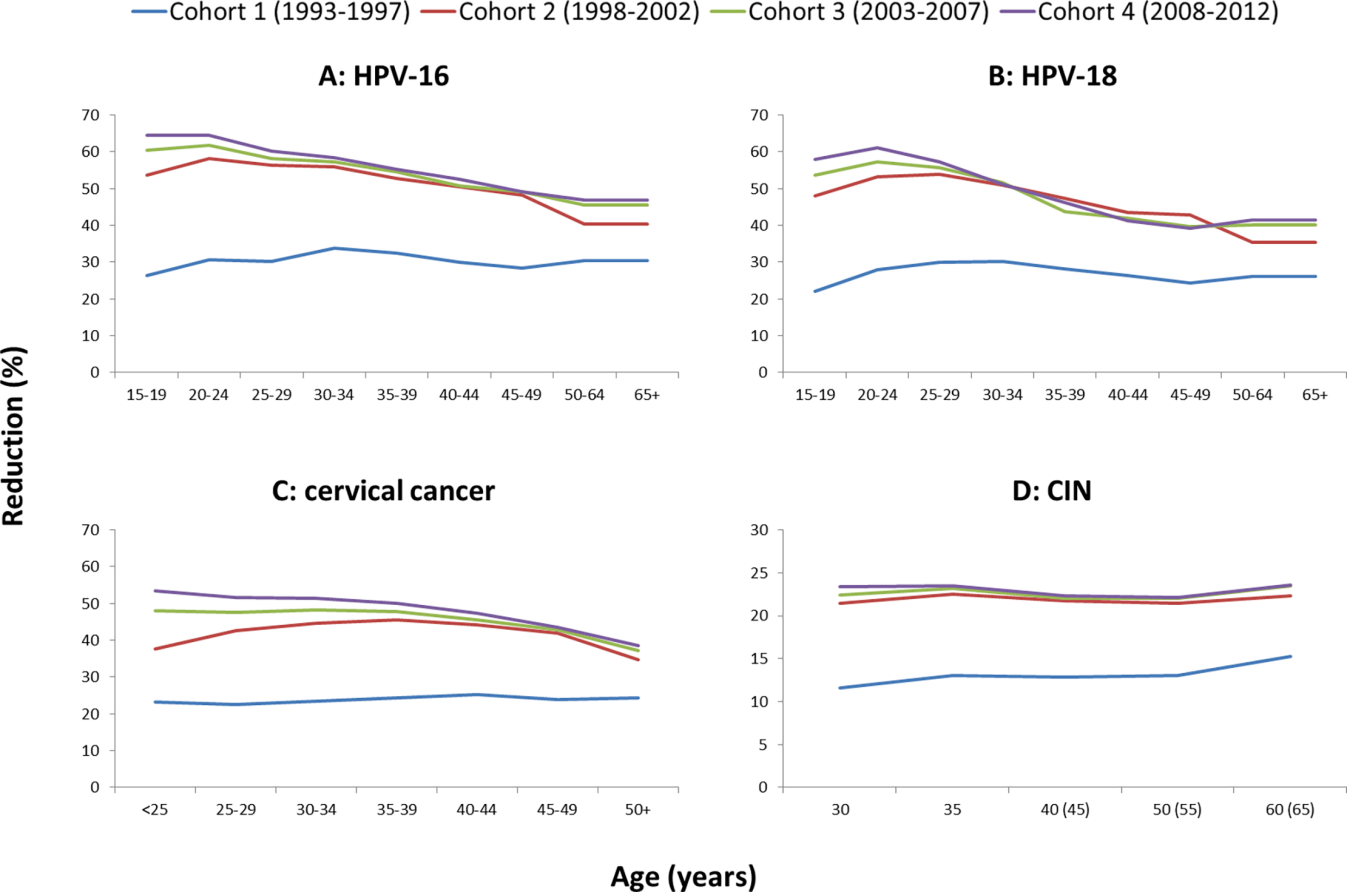
**Relative reductions by age group in the incidence of HPV-16 (A), HPV-18 (B), cervical cancer (C), and CIN (D) for the first four successive 5-year birth cohorts (vaccinated and unvaccinated women) that underwent the current girls-only vaccination program, compared to no vaccination.** Cohort 1 is born between 1993–1997; cohort 2 between 1998–2002; cohort 3 between 2003–2007; and cohort 4 between 2008–2012. The ages in parentheses on the x-axis of Fig 1D depict the additional screen ages 45 and 55 if women did not attend screening or tested HPV positive at ages 40 and 50, respectively, and at age 65 if women attended and tested positive at age 60.

Fig 1 shows the estimated relative reductions in the incidence of HPV-16, HPV-18, cervical cancer, and diagnosed CIN per birth cohort under the current vaccination program compared to no vaccination. For all cohorts, the relative incidence reductions were larger for HPV-16 than HPV-18 infections. The younger the birth cohort, the larger the health impact of HPV vaccination. The larger reductions in HPV incidence and corresponding age patterns for younger cohorts are attributable to higher levels of herd immunity, as HPV transmission reduces over time. Also, almost all girls in cohort 1 have been offered HPV vaccination by means of the catch-up campaign given their age, which had a lower coverage rate than the annual vaccination of 12-year-old girls (i.e. 50% compared to 60%). Cohort 1 therefore has relatively fewer vaccinated girls than the younger cohorts, as the younger cohorts all received vaccination according to the annual vaccination of 12-year-old girls. While for the younger cohorts a trend by age is visible (ranging from over 50% in cervical cancer incidence for women younger than 25 years to almost 40% for women aged 65+), the reductions for the oldest cohort (cohort 1) are quite stable over all ages (between 22% and 25% reduction over all ages). A slight increase in the reduction of CIN lesions is visible at the last screening age. As the percentage of CIN attributable to HPV-16 and HPV-18 slightly increase with age in the model, the impact of the vaccine on CIN is larger at these ages.

Our models predict that the HPV-16 and HPV-18 incidence reductions will lead to a 35% lifetime reduction in clinically detected cancers and cervical cancer deaths, and almost 40% in screen-detected cancers, compared to no vaccination (Table 1). Somewhat larger health gains are accomplished when coverage of HPV vaccination among girls increases to 80% and 100%, i.e. 43% reduction in cervical cancer cases and cancer deaths under 80% coverage, and over 50% reduction in cancer cases and deaths under full coverage. However, even when vaccine uptake among girls can be increased from 60% to 80%, the health gains are still lower as compared to expanding the target group to males. Including males by offering adult vaccination at STI clinics for both sexes (which also incorporates routine boys vaccination) and solely including routine boys vaccination with current uptake levels are predicted to reduce the number of cervical cancer deaths by 49% and 45%, respectively, as compared to 43% when coverage among girls is increased to 80%. Including boys with increased uptake of 80% for both sexes leads to larger reductions in cervical cancer cases (54%) and deaths (56%) than full coverage among girls (51% and 52%, respectively). As expected, the largest health gains are accomplished when full coverage can be achieved for routine girls and boys vaccination, with reductions of 61% and 64% in cancer cases and deaths, respectively.

**Table 1:** Health impact of the current girls-only vaccination program and alternative vaccination strategies on cervical disease per 100,000 women. The relative lifetime change as compared to no vaccination are shown between parentheses. In the alternative vaccination strategies, boys, and adult women and men are included in the vaccination strategies in addition to girls. Vaccination at STI consultations for adult males and females also includes routine vaccination for boys.

Strategy	False-positive referrals	CIN 1	CIN 2	CIN 3	Clinically detected cases	Screen-detected cancers	Cervical cancer deaths	Life years lost
No vaccination	543	2,473	1,655	2,413	444	136	193	3,089
Current program (60% cov.)	458 (-16%)	2,121 (-14%)	1,361 (-18%)	1,778 (-26%)	289 (-35%)	83 (-39%)	126 (-34%)	1,979 (-36%)
*Improved coverage among girls*								
Girls (80% cov.)	448 (-17%)	2,061 (-17%)	1,318 (-20%)	1,696 (-30%)	256 (-42%)	76 (-44%)	110 (-43%)	1,772 (-43%)
Girls (100% cov.)	431 (-21%)	2,006 (-19%)	1,276 (-23%)	1,619 (-33%)	215 (-52%)	70 (-49%)	92 (-52%)	1,555 (-50%)
*Inclusion of also boys and adults*								
Boys (60% cov.)	443 (-18%)	2,037 (-18%)	1,310 (-21%)	1,682 (-30%)	246 (-45%)	74 (-46%)	105 (-45%)	1,715 (-44%)
Boys (80% cov.)	430 (-21%)	1,976 (-20%)	1,258 (-24%)	1,596 (-34%)	201 (-55%)	67 (-51%)	85 (-56%)	1,469 (-52%)
Boys (100% cov.)	416 (-23%)	1,922 (-22%)	1,222 (-26%)	1,526 (-37%)	168 (-62%)	60 (-56%)	70 (-64%)	1,285 (-58%)
STI consultations (F)	454 (-16%)	2,093 (-15%)	1,336 (-19%)	1,732 (-28%)	274 (-38%)	80 (-42%)	119 (-38%)	1,885 (-39%)
STI consultations (F+M)	438 (-19%)	2,004 (-19%)	1,286 (-22%)	1,631 (-32%)	231 (-48%)	70 (-49%)	98 (-49%)	1,621 (-48%)

CIN = cervical intraepithelial neoplasia; cov. = coverage; STI = sexually transmitted infections; F = females; M = males

**Fig 2:**
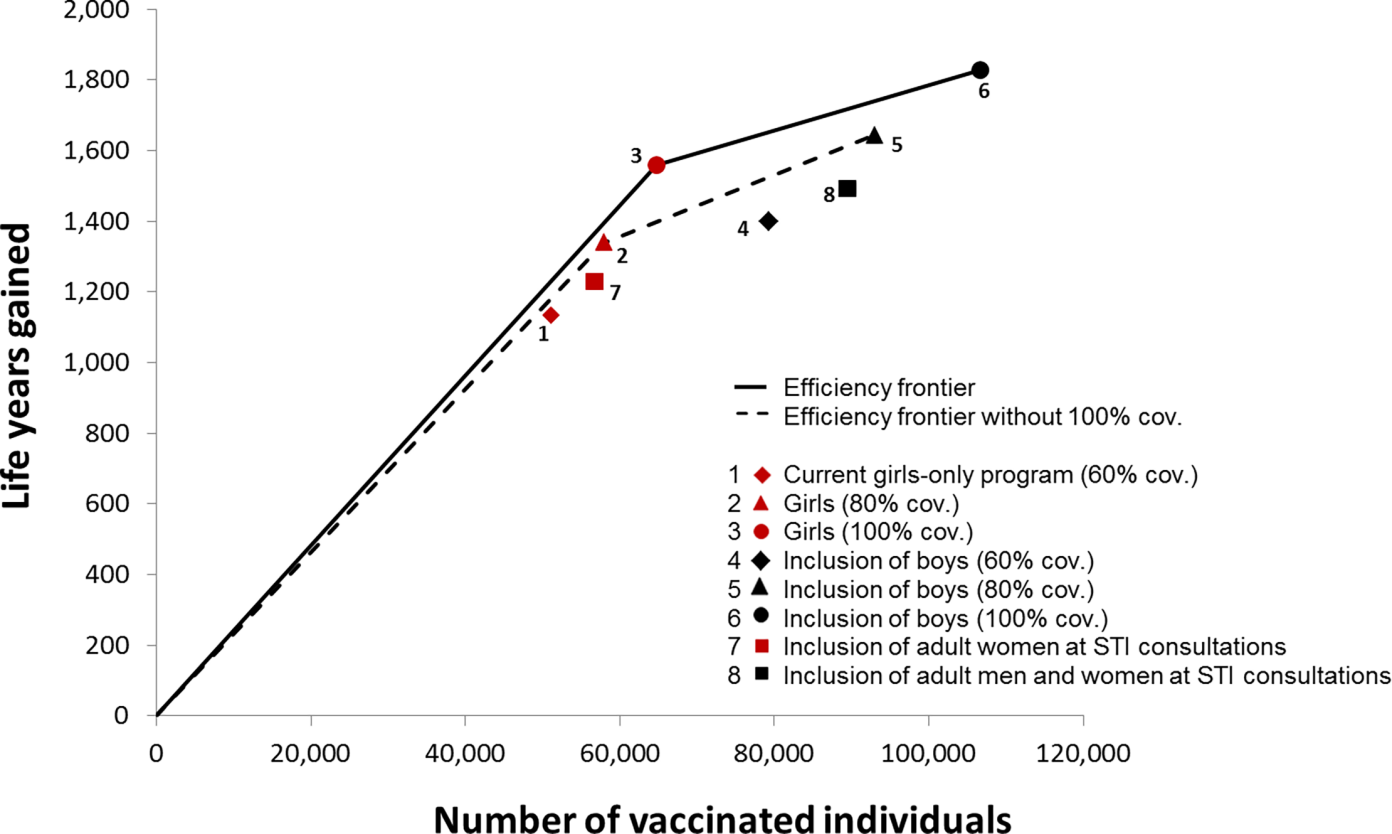
Estimated number of life years gained and corresponding number of vaccinated individuals for the current vaccination program and alternative strategies as compared to no vaccination program. The current vaccination program has been implemented in 2009. The alternative vaccination strategies commence from 2017 onward, in addition to the current program. Numbers are scaled to a population of 100,000 women in 2017. Cov. = coverage; STI = sexually transmitted infection.

Under the current vaccination program, vaccinating 45 girls (NNV = 51,070/1,134) will save one life year by preventing cervical cancer death, when compared to no vaccination (Fig 2). Most efficient strategies are: achieving full coverage among girls (NNV = 42); and achieving full coverage of routine vaccination for both sexes (incremental NNV = 155). Less efficient are routine vaccination for both sexes with 60% and 80% coverage, and vaccination during STI consultations for both sexes. When the strategies with full coverage are excluded from the analyses, improving coverage to 80% in girls and both sexes become the most efficient strategies.

The sensitivity analyses in Table 2 show that, while the absolute disease burdens with and without vaccination differ, the relative health impact of the current vaccination program is nearly similar under alternative levels of cervical cancer screening attendance and with a higher baseline HPV prevalence. The largest difference in health impact between the base case and sensitivity analyses was found when we assumed a higher baseline HPV prevalence, and there was only a 3 percentage points difference in the relative reduction in cervical cancer deaths and life years lost.

**Table 2:** Health impact of the current girls-only vaccination program under alternative levels of cervical cancer screening attendance and with doubled baseline HPV prevalence. Results are shown per 100,000 women. The relative change as compared to no vaccination are shown between parentheses. Alternative levels of attendance include either 20% higher or lower than the observed attendance in the current screening program.

	False-positive referrals	CIN 1	CIN 2	CIN 3	Clinically detected cases	Screen-detected cancers	Cervical cancer deaths	Life years lost
*Base case*								
No vaccination	543	2,473	1,655	2,413	444	136	193	3,089
Current program	458 (-16%)	2,121 (-14%)	1,361 (-18%)	1778 (-26%)	289 (-35%)	83 (-39%)	126 (-34%)	1,979 (-36%)
*Higher attendance*								
No vaccination	637	2,836	1,896	2,647	389	121	174	2,542
Current program	531 (-17%)	2,438 (-14%)	1,556 (-18%)	1,961 (-26%)	256 (-34%)	72 (-40%)	115 (-34%)	1,619 (-36%)
*Lower attendance*								
No vaccination	451	2,059	1,371	2,097	566	144	240	4,222
Current program	379 (-16%)	1,765 (-14%)	1,129 (-18%)	1,536 (-27%)	362 (-36%)	88 (-39%)	156 (-35%)	2,666 (-37%)
*Higher baseline HPV prevalence*								
No vaccination	950	2,528	1,679	2,423	446	137	195	3,185
Current program	808 (-15%)	2,172 (-14%)	1,375 (-18%)	1,786 (-26%)	285 (-36%)	82 (-40%)	123 (-37%)	1,950 (-39%)

CIN = cervical intraepithelial neoplasia; HPV = human papillomavirus; vacc. = vaccination

## Discussion

Using a comprehensive modeling framework, linking the established STDSIM and MISCAN-Cervix models, we have estimated the health impact of the current girls-only vaccination program and various alternative vaccination strategies under the new cervical cancer screening program in the Netherlands. HPV-16 and HPV-18 incidence reductions achieved under the current girls-only vaccination are predicted to lead to substantial reductions in cervical disease burden, with an estimated reduction of 35% in clinically detected cancers and cervical cancer deaths, and almost 40% in screen-detected cancers, compared to no vaccination. The NNV of the current girls-only program is 45. Largest health gains will be accomplished when full coverage can be achieved for routine vaccination of both girls and boys. For this strategy, the incremental NNV to gain one life year is 155, compared to the most efficient strategy of full coverage among girls (NNV = 42).

The larger reductions in HPV incidence and corresponding age patterns for younger cohorts are attributable to higher levels of herd immunity. This was also demonstrated in the study of Bogaards *et al*., in which they concluded that the reduction of hazard in unvaccinated women increases with birth cohort year [43]. For even younger cohorts than the ones included in our study, the HPV incidence reductions will be more substantial than in the youngest simulated birth cohort, and will therefore have more health gains due to vaccination.

We have estimated the reduction in cervical cancer due to HPV vaccination before, but in a crude way [7]. In the current study, the estimated reduction in (screen- and clinically detected) cancers is smaller than the incidence reduction we found in our earlier study under the current vaccination program (36% versus 48%, respectively). Three distinctions between the studies are important to consider when interpreting this difference. First, in the previous study we used a relatively simple calculation for the reduction in cancers instead of a detailed microsimulation model. Second, primary screening methods differ between both studies. While the previous study uses incidence data from a population with primary cytology screening, we simulated here the new program with primary HPV screening, which is expected to prevent more cervical cancer cases itself [10]. Third, in the previous study, we used the incidence reduction when a steady state is achieved (approximately 70 years following the introduction of HPV vaccination), while here we estimated the lifetime health impact of the four earliest vaccinated cohorts. As the impact of HPV vaccination on HPV incidence has not reached its full potential prior to the steady state, the cancer incidence reductions are smaller than for younger, i.e. steady state, birth cohorts.

Including also routine vaccination for boys with 80% coverage was predicted to prevent more deaths than reaching full coverage among girls (56% reduction versus 53%). However, including boys appears less efficient, due to a larger increase in number of vaccinations needed. We appreciate that full coverage of the vaccination program, either for only girls or both sexes, might be unrealistic. We therefore also explored the efficiency of the vaccination strategies when excluding the scenarios of full coverage (Fig 2). In that situation the most efficient strategies include increasing coverage to 80% for girls (NNV = 43) and for both sexes (incremental NNV = 115). These results indicate that including boys is an efficient strategy to further improve cervical disease prevention, and that the higher the uptake, the more efficient this strategy will be. Finally, our model shows that vaccination offered at STI consultations is least efficient, likely due to the fact that people attending STI consultations have already been exposed to HPV, and are more likely to either be infected with HPV, or be immune due to clearance of an earlier infection, at the moment of vaccination.

Even though the models are quantified to data from the Netherlands, these results are generalizable to developed settings with a higher prevalence of HPV or cervical disease. While a higher prevalence of HPV-related cervical disease would lead to a higher absolute number of referrals and treatments, the relative reductions due to vaccination will not change substantially, as long as the proportion of cervical disease attributable to HPV-16 and -18 remains the same. This is supported by the sensitivity analysis with the higher HPV baseline prevalence (by increasing the prevalence of transient infections), in which indeed absolute numbers differ from those in the base case analysis, yet the relative reductions due to vaccination are similar.

Our study has four limitations that are noteworthy. First, in our model, we underestimated the proportion of CIN 2 attributable to HPV-16, and slightly overestimated the proportion of CIN 1 and CIN 3 attributable to HPV-18, compared to the observed proportions [30]. These discrepancies are small though, and less relevant than the associated cancer cases and deaths, which were estimated well. Also, they apply to all strategies, so that the comparative effects of different scenarios are still of value. Second, it is still uncertain if the attendance rate of the screening program will be influenced by the switch to primary HPV screening and the opt-in procedure of the self-sampling kit. However, our sensitivity analyses showed that alternative attendance rates do not influence the health impact estimates substantially. Third, our estimates of the health impact are conservative, as we did not take cross-protection of the bivalent vaccine or additional protective effects of the nonavalent vaccine into account. Finally, we compared vaccination strategies based on the NNV to gain one life year, while policy decisions are usually based on costs per life year gained. The NNV measure, however, is easy to interpret and does allow for comparing vaccination strategies in terms of their efficiency to achieve the main goal of HPV vaccination programs (i.e. reduce cervical cancer mortality). In a full cost-effectiveness analysis, one would not only need to include the costs and benefits of HPV vaccination and cervical cancer screening and treatment, but also those related to all other HPV-related diseases, including e.g. also genital warts. This was beyond the scope of the current work, but would be an interesting topic for further research.

We conclude that, already for the first vaccinated birth cohorts, the current vaccination program will lead to substantial reductions in cervical cancer incidence and mortality in the Netherlands. Efficient strategies to further improve health gains are to increase vaccination uptake among girls, or to extend the target group to routine boys vaccination with increased vaccination uptake. As vaccination will not prevent all cervical cancer deaths, screening does not become obsolete and participation should continue to be encouraged in the post-vaccination era.

## Supporting information

S1 Text MISCAN-Cervix model profile. (DOCX)

S1 Fig Structure of the MISCAN-Cervix model. (TIF)

S2 Fig Schematic representation of the MISCAN-cervix model, with disease pathways A through F. There are six disease pathways (types A through F) in MISCAN. All lesions start as either an HPV infection without CIN (disease pathways A, B, C, D, and F) or as a CIN 1 lesion without HPV infection (disease pathway E). Cleared/regressed denotes the absence of CIN and HPV infection; CIN 0 denotes the absence of CIN and cervical cancer. All cervical cancer states are HPV positive. The arrows between the states show which types of transitions can occur; the numbers refer to the duration distributions shown in S4 Table. In every state before death, a transition to “Other-cause death” can occur, and in every state before cancer, a transition to “Hysterectomy” can occur (connecting arrows not shown); in these cases, the transition applies to all HPV infections and CIN lesions of that person simultaneously.
(TIF)

S3 Fig Annual probability of acquiring a regressive HPV infection or CIN lesion (top graph) and annual probability of acquiring a progressive HPV infection (bottom graph).
(TIF)

S4 Fig Relative reductions in HPV-16 and HPV-18 incidence for the first four successive 5-year birth cohorts (vaccinated and unvaccinated women) that were offered the current girls-only vaccination program estimated by STDSIM, applied to the progressive pathway (infections leading to invasive cancer) in MISCAN-Cervix. Cohort 1 is born between 1993–1997; cohort 2 between 1998–2002; cohort 3 between 2003–2007; and cohort 4 between 2008–2012.
(TIF)

S1 Table Age-specific probability of having had a hysterectomy for reasons other than cervical cancer. Linear interpolation is used to determine the probability of having had a hysterectomy at intermediate ages.
(DOCX)

S2 Table Cervical cancer incidence by age and FIGO stage. (DOCX)

S3 Table **Age-specific test results and histological diagnoses of cervical cancer screening in the Netherlands in years 2000–2007, for (A) women’s first lifetime screen and (B) subsequent screens.** Data were obtained from the nationwide registry of histo- and cytolopathology in the Netherlands (PALGA).
(DOCX)

S4 Table Transitions and duration distributions used in MISCAN-Cervix. (DOCX)

S5 Table Age-specific probability that cervical cancer is detected in stages FIGO 1B and FIGO 2+, given that it is clinically detected. Percentages in the table are estimated in the model calibration. Linear interpolation is used to determine the probabilities at intermediate ages.
(DOCX)

S6 Table Model assumptions for the age-specific probability that clinical FIGO 1B and FIGO 2+ cervical cancer will lead to death from cervical cancer (i.e. 100%—probability of long-term survival), in the absence of other-cause mortality. Linear interpolation is used to determine the probabilities at intermediate ages. Source: observed age-specific and stage-specific survival for the periods 1989–2002 and 2003–2009, obtained from the Dutch Cancer Registry.
(DOCX)

S7 Table Model assumptions for the duration distribution of clinical FIGO 1B and FIGO 2+ cervical cancer, if the transition to death from cervical cancer occurs. The values in this table represent the percentages of cervical cancer deaths that occur within a given number of years after the moment of clinical diagnosis. It is assumed that no cervical cancer mortality occurs more than 10 years after clinical diagnosis. Source: observed age-specific and stage-specific survival for the periods 1989–2002 and 2003–2009, obtained from the Dutch Cancer Registry.
(DOCX)

S8 Table The observed and estimated proportions of HPV-16 and HPV-18 in HPV infections without cytological abnormalities, and in CIN 1, CIN 2, CIN 3, and invasive cervical cancer in the population prior to vaccination in MISCAN-Cervix. The observed proportions are based on the studies of Coupé *et al*., who reported age-specific HPV prevalence in women aged 18–65 years, and Guan *et al*. who determined the distribution of HPV types in CIN and cervical cancer (large meta-analysis of studies with different age ranges)[30, 31]. HPV = human papillomavirus; CIN = cervical intraepithelial neoplasia.
(DOCX)

S9 Table Attendance in the cervical cancer screening program and self-sampling kit in the base case and sensitivity analyses, based on the observed screening attendance in 2013[36]. In the model, we assumed that 10% of women never attend screening at the general practitioner, and that 90% are potential attenders[29]. Of the non-attending women, 3% opt-in to receive a self-sampling kit. *Attendance at age 65 is assumed to be equal to the observed attendance at age 60.
(DOCX)
