# Supplementary material for: The health impact of human papillomavirus vaccination in the situation of primary human papillomavirus screening: A mathematical modeling study
Source: PLoS One. 2018 Sep 4;13(9):e0202924. doi: 10.1371/journal.pone.0202924 (PMC6122803; doi:10.1371/journal.pone.0202924)
Supplement: S1 Text — (DOCX) [file pone.0202924.s001.docx]

**MISCAN-Cervix model profile**

In this appendix, we describe the model inputs of the Microsimulation Screening Analysis (MISCAN) model for cervical cancer [1]. This model can be used to assess the harms and benefits of different screening programs for cervical cancer, as well as human papillomavirus (HPV) vaccination. The model has been used previously for cost-effectiveness analyses of cervical cancer screening and HPV vaccination [2-6]. Sections below have in nearly identical form also been published in Naber *et al.*[6]

**Model structure**

S1 Fig shows the structure of MISCAN-Cervix. The model consists of the following 4 parts: demography, natural history, screening, and effectiveness. The assumptions used in each of these parts are described below.

**Demography**

The MISCAN-Cervix model generates a simulated population of women, of which general characteristics of the simulated population (i.e. those not related to the disease) are based on demographic and hysterectomy data. The assumed hysterectomy rates vary by age. These rates from 2010 are calculated using data from Statistics Netherlands and the Information Centre for Health Care (S1 Table)[7, 8]. For each woman, a time of death from other causes than cervical cancer is generated; this time of death is independent of the cervical cancer disease model. In the model, a woman’s lifetime cannot exceed 100 years. The time of death from other causes is generated using the life table of 2014 for women from Statistics Netherlands[8].

**Natural history**

During her lifetime, each woman has an age-specific risk of acquiring high-risk HPV infections (i.e. an infection caused by an HPV type that can cause cancer and that can potentially be detected by the HPV test) and CIN lesions without a (detectable) high-risk HPV infection (i.e. HPV negative CIN lesions). These high-risk infections include HPV-16, HPV-18, and other high-risk HPV infections. Most HPV infections clear or regress naturally, some HPV infections can progress to CIN 1, CIN 2, CIN 3, cervical cancer, and death from cervical cancer.

The age-specific incidence of HPV infections that progress to cervical cancer was calibrated to the age-specific incidence of cervical cancer, which was obtained from the Dutch Cancer Registry (S2 Table). The age-specific incidence of pre-invasive lesions that do not progress to cervical cancer was calibrated so that the simulated detection rates of CIN lesions fit the observed detection rates in the Netherlands. The observed detection rates were obtained from the Dutch Network and National Database for Pathology (PALGA) for the period 2000-2007 (S3 Table)[9]. The incidence of high-risk HPV infections that do not progress to CIN was calibrated so that the simulated prevalence of all high-risk HPV infections fits the observed high-risk HPV prevalence[10, 11].

In MISCAN-Cervix, 6 disease pathways are distinguished. Each instance of these disease pathways represents an HPV infection or a ‘lesion’ (i.e. CIN of a certain grade or a stage of cervical cancer). Each disease pathway starts as either an HPV infection or as an HPV negative CIN 1 lesion. The natural history (i.e. in the situation without screening) of these 6 disease pathways is shown in S2 Fig and can be described as follows:

A) HPV infections that clear naturally without ever leading to CIN

B) HPV infections that progress to CIN 1 and then regress

C) HPV infections that progress to CIN 1 and CIN 2 and then regress

D) HPV infections that progress to CIN 1, CIN 2, and CIN 3 and then regress

E) HPV negative CIN 1 lesions that regress naturally or become HPV negative CIN 2 and then regress naturally

F) HPV infections that progress to CIN 1, CIN 2, CIN 3, preclinical FIGO 1A (micro-invasive) cervical cancer, and preclinical FIGO 1B cervical cancer. Preclinical FIGO 1B cervical cancer can either become clinically detected FIGO 1B cervical cancer or progress to preclinical FIGO 2+ cervical cancer and then to clinical FIGO 2+ cervical cancer. Clinically detected cervical cancer can progress to death from cervical cancer or remain in that state forever (if the woman is cured from cervical cancer).

A woman can acquire multiple lesions and HPV infections during her lifetime, and multiple lesions and HPV infections may be present at the same time. In each simulated life history, the number of lesions follows a Poisson distribution. The annual probability of acquiring an HPV infection or CIN lesion is age-dependent and depicted in S3 Fig. The transitions and sojourn times of the HPV infections or lesions are simulated based on a continuous-time semi-Markov process. The sojourn times of most states in the model have either an exponential or a Weibull probability distribution (S4 Table).

In the model, women who do not have cervical cancer have an age-specific probability of getting a hysterectomy for reasons other than cervical cancer. A hysterectomy is assumed to remove all prevalent HPV infections and CIN lesions. Women with a hysterectomy will no longer acquire HPV infections or CIN lesions and are also no longer invited for screening tests.

The assumptions for the probability and the duration of survival after a clinically detected (i.e. detected because of symptoms) cervical cancer are based on data from the Dutch Cancer Registry for the period 1989-2009 [6]. As these data include both adenocarcinoma and squamous cell carcinoma, the survival we estimated is a weighted average of these two types of cervical cancer. We assumed that all cervical cancer mortality occurs in the first 10 years after diagnosis. The assumed probability of long term survival depends on age and stage (FIGO 1B or FIGO 2+); in the model, FIGO 1A cervical cancer cannot be clinically detected. S5 Table shows what percentage of clinically detected cancers is detected in stages FIGO 1B and FIGO 2+. The model assumptions for the long-term survival probabilities are shown in S6 Table and the assumed duration distributions are shown in S7 Table.

**Screening**

Screening can change the life histories of women. In the current analyses, we simulated the new primary HPV screening program in the Netherlands, as described in the main manuscript. In the model, detection of cervical cancer by screening prevents death from cervical cancer in some but not all cases. However, if death from cervical cancer is not prevented, the time of death from cervical cancer is not changed by screening.

For screen-detected invasive cancers, survival was modeled as a reduction in the risk of dying compared with that risk in the situation without screening, when the cancer would have become clinical. This improvement of prognosis (89.4%, 50% and 20% for FIGO 1A, 1B and 2+ respectively) was calibrated to reproduce recently observed stage specific survival given observed screening (Dutch Cancer Registry)[6].

**Effectiveness**

For each simulated woman who is alive, MISCAN-Cervix can determine the state, which can be Normal, HPV infected, CIN 1, CIN 2, CIN 3, FIGO 1A, FIGO 1B, and FIGO 2+. A woman can have multiple HPV infections or CIN lesions at the same time. Her state is determined by the most severe disease stage present, using the order HPV infection, CIN 1, CIN 2, CIN 3, FIGO 1A cervical cancer, FIGO 1B cervical, and FIGO 2+ cervical cancer; if no HPV infections or CIN lesions are present, the woman’s state is Normal. The model produces the number of life years spent in each state as well as the number of certain events (e.g. screenings and cervical cancer diagnoses) in a lifetime.

**References**

1. Habbema JD, Van Oostmarssen G, Lubbe JT, Van der Maas PJ. The MISCAN simulation program for the evaluation of screening for disease. Comput Methods Programs Biomed. 1985;20(79-93).

2. de Kok IM, van Ballegooijen M, Habbema JD. Cost-effectiveness analysis of human papillomavirus vaccination in the Netherlands. J Natl Cancer Inst. 2009;101(15):1083-92. Epub 2009/07/03. doi: djp183 [pii]

10.1093/jnci/djp183. PubMed PMID: 19571256.

3. van den Akker-van Marle ME, van Ballegooijen M, van Oortmarssen GJ, Boer R, Habbema JD. Cost-effectiveness of cervical cancer screening: comparison of screening policies. J Natl Cancer Inst. 2002;94(3):193-204. Epub 2002/02/07. PubMed PMID: 11830609.

4. de Kok IM, van Rosmalen J, Dillner J, Arbyn M, Sasieni P, Iftner T, et al. Primary screening for human papillomavirus compared with cytology screening for cervical cancer in European settings: cost effectiveness analysis based on a Dutch microsimulation model. BMJ. 2012;344:e670. Epub 2012/03/07. PubMed PMID: 22391612; PubMed Central PMCID: PMC3293782.

5. van Rosmalen J, de Kok IM, van Ballegooijen M. Cost-effectiveness of cervical cancer screening: cytology versus human papillomavirus DNA testing. BJOG. 2012;119(6):699-709. Epub 2012/01/19. doi: 10.1111/j.1471-0528.2011.03228.x. PubMed PMID: 22251259; PubMed Central PMCID: PMC3489039.

6. Naber SK, de Kok IM, Matthijsse SM, van Ballegooijen M. The potential harms of primary human papillomavirus screening in over-screened women: a microsimulation study. Cancer Causes Control. 2016. Epub 2016/03/14. doi: 10.1007/s10552-016-0732-7

10.1007/s10552-016-0732-7 [pii]. PubMed PMID: 26970740.

7. SIG (Information Centre for Health Care). Hospital Diagnosis Statistics 1963-1985. Utrecht: SIG.

8. Statistics Netherlands. Statline database population key figures. (March 2012).

9. Casparie M, Tiebosch AT, Burger G, Blauwgeers H, Van de Pol A, Van Krieken JH. Pathology databanking and biobanking in The Netherlands, a central role for PALGA, the nationwide histopathology and cytopathology data network and archive. Cell Oncology. 2007;29(1):19-24.

10. Bulkmans NW, Rozendaal L, Snijders PJ, Voorhorst FJ, Boeke AJ, Zandwijken GR, et al. POBASCAM, a population-based randomized controlled trial for implementation of high-risk HPV testing in cervical screening: design, methods and baseline data of 44,102 women. Int J Cancer. 2004;110(1):94-101. PubMed PMID: 15054873.

11. Lenselink CH, Melchers WJ, Quint WG, Hoebers AM, Hendriks JC, Massuger LF, et al. Sexual behaviour and HPV infections in 18 to 29 year old women in the pre-vaccine era in the Netherlands. PLoS One. 2008;3(11):e3743. Epub 2008/11/18. doi: 10.1371/journal.pone.0003743. PubMed PMID: 19011683; PubMed Central PMCID: PMC2581437.
