# Supplementary figures and images for: The health impact of human papillomavirus vaccination in the situation of primary human papillomavirus screening: A mathematical modeling study
Source: PLoS One. 2018 Sep 4;13(9):e0202924. doi: 10.1371/journal.pone.0202924 (PMC6122803; doi:10.1371/journal.pone.0202924)

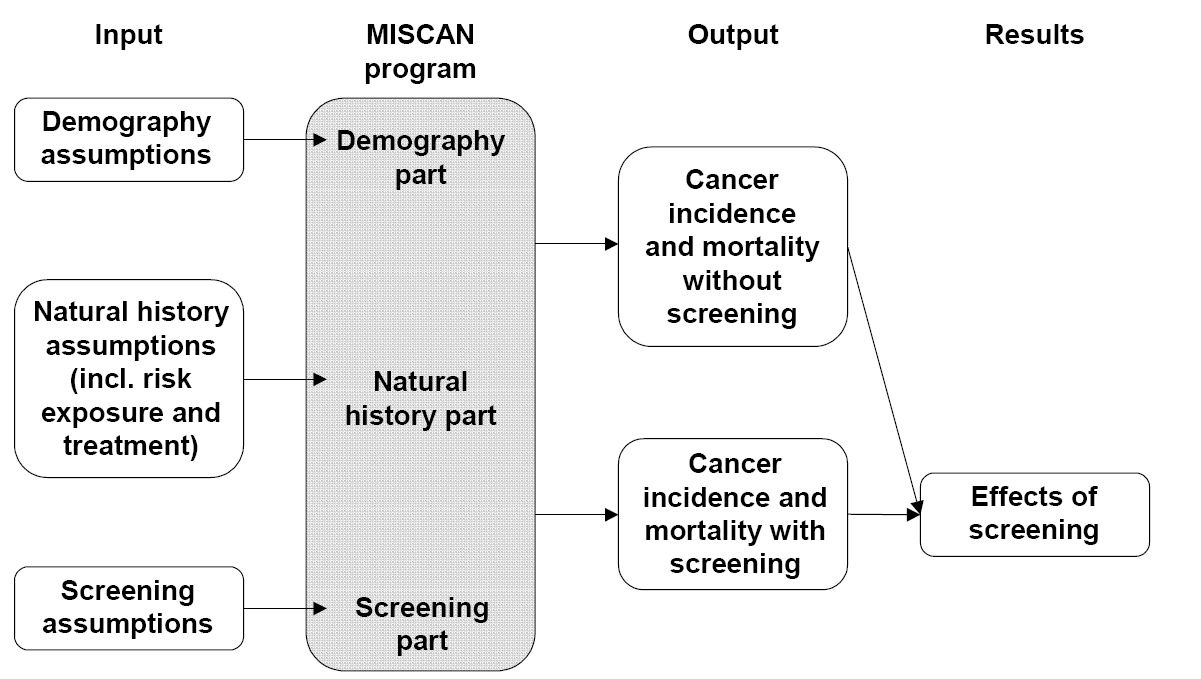

Supplement: S1 Fig — (TIF) [file pone.0202924.s002.tif]

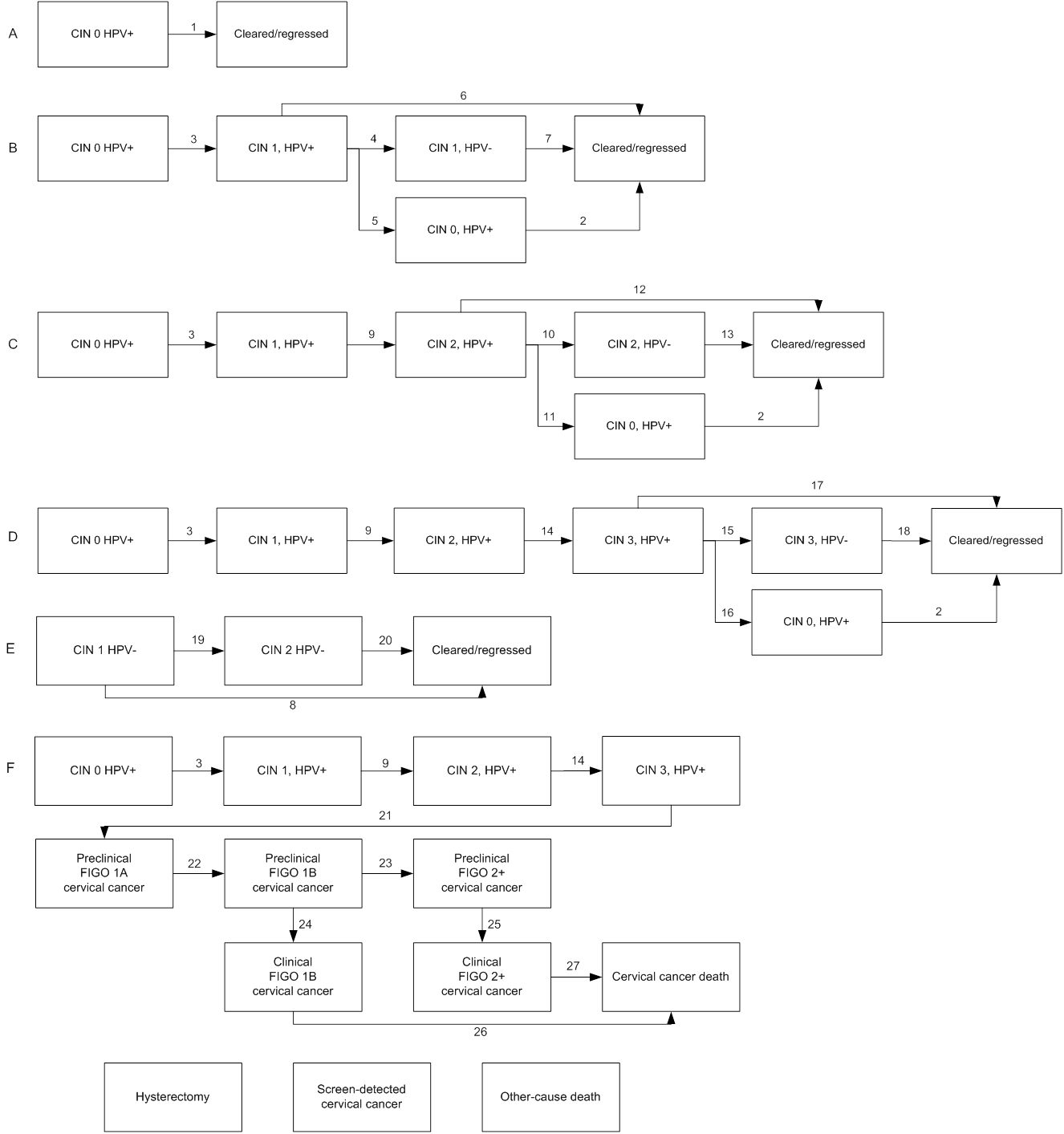

Supplement: S2 Fig — There are six disease pathways (types A through F) in MISCAN. All lesions start as either an HPV infection without CIN (disease pathways A, B, C, D, and F) or as a CIN 1 lesion without HPV infection (disease pathway E). Cleared/regressed denotes the absence of CIN and HPV infection; CIN 0 denotes the absence of CIN and cervical cancer. All cervical cancer states are HPV positive. The arrows between the states show which types of transitions can occur; the numbers refer to the duration distributions shown in S4 Table. In every state before death, a transition to “Other-cause death” can occur, and in every state before cancer, a transition to “Hysterectomy” can occur (connecting arrows not shown); in these cases, the transition applies to all HPV infections and CIN lesions of that person simultaneously. (TIF) [file pone.0202924.s003.tif]

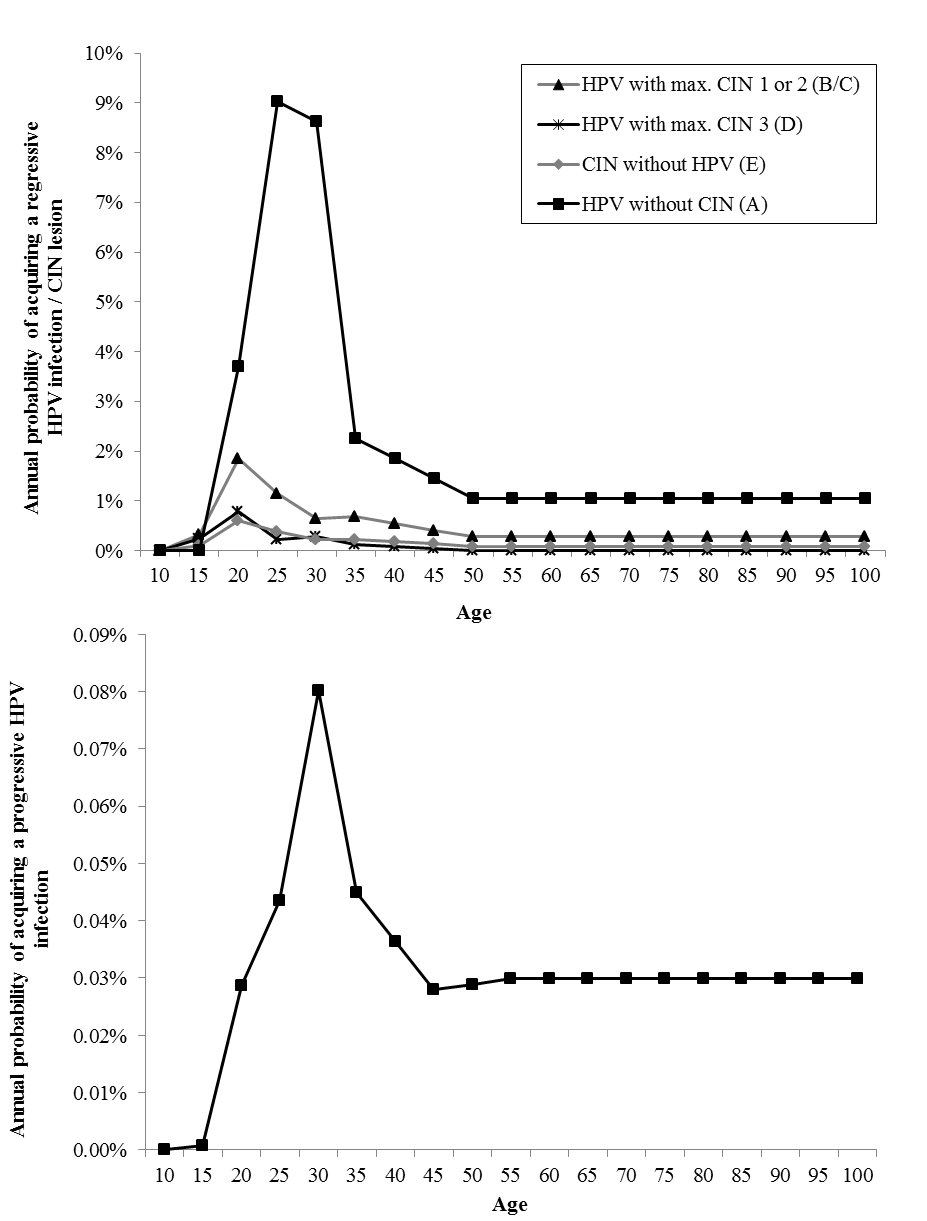

Supplement: S3 Fig — Annual probability of acquiring a regressive HPV infection or CIN lesion (top graph) and annual probability of acquiring a progressive HPV infection (bottom graph). (TIF) [file pone.0202924.s004.tif]

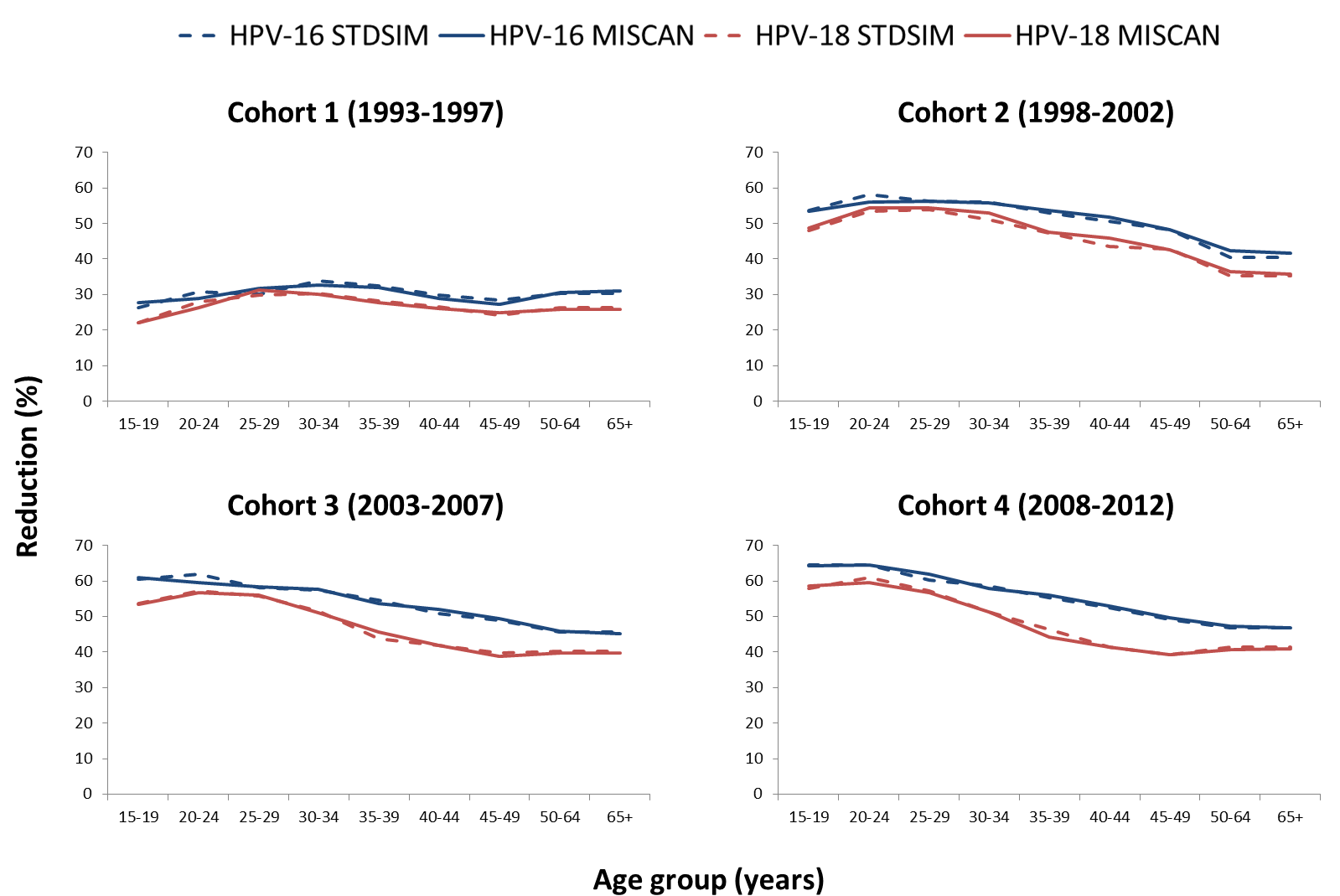

Supplement: S4 Fig — Cohort 1 is born between 1993–1997; cohort 2 between 1998–2002; cohort 3 between 2003–2007; and cohort 4 between 2008–2012. (TIF) [file pone.0202924.s005.tif]
