# Supplementary material for: The health impact of human papillomavirus vaccination in the situation of primary human papillomavirus screening: A mathematical modeling study
Source: PLoS One. 2018 Sep 4;13(9):e0202924. doi: 10.1371/journal.pone.0202924 (PMC6122803; doi:10.1371/journal.pone.0202924)
Supplement: S1 Table — Linear interpolation is used to determine the probability of having had a hysterectomy at intermediate ages. (DOCX) [file pone.0202924.s006.docx]

**S1 Table. Age-specific probability of having had a hysterectomy for reasons other than cervical cancer.** Linear interpolation is used to determine the probability of having had a hysterectomy at intermediate ages.

| **Age** | **Cumulative probability of having had a hysterectomy** |
| --- | --- |
| 20 | 0.0000 |
| 25 | 0.0002 |
| 30 | 0.0017 |
| 35 | 0.0076 |
| 40 | 0.0213 |
| 45 | 0.0432 |
| 50 | 0.0735 |
| 55 | 0.0916 |
| 60 | 0.1009 |
| 65 | 0.1102 |
| 70 | 0.1217 |
| 75 | 0.1330 |
| 80 | 0.1419 |
| 85 | 0.1468 |
