# Supplementary material for: The health impact of human papillomavirus vaccination in the situation of primary human papillomavirus screening: A mathematical modeling study
Source: PLoS One. 2018 Sep 4;13(9):e0202924. doi: 10.1371/journal.pone.0202924 (PMC6122803; doi:10.1371/journal.pone.0202924)
Supplement: S2 Table — (DOCX) [file pone.0202924.s007.docx]

**S2 Table. Cervical cancer incidence by age and FIGO stage.**

|  | **FIGO stage** | | | | | | | | | | | | | |
| --- | --- | --- | --- | --- | --- | --- | --- | --- | --- | --- | --- | --- | --- | --- |
| **Age** | | I | Ia | Ib | II | IIa | IIb | III | IIIa | IIIb | IVa | IVb | unknown | total |
| 1 | | 0 | 0 | 0 | 0 | 0 | 0 | 0 | 0 | 0 | 0 | 0 | 0 | 0 |
| 2 | | 0 | 0 | 0 | 0 | 0 | 0 | 0 | 0 | 0 | 0 | 0 | 0 | 0 |
| 3 | | 0 | 0 | 0 | 0 | 0 | 0 | 0 | 0 | 0 | 0 | 0 | 0 | 0 |
| 4 | | 0 | 0 | 0 | 0 | 0 | 0 | 0 | 0 | 0 | 0 | 0 | 0 | 0 |
| 5 | | 0 | 0 | 0 | 0 | 0 | 0 | 0 | 0 | 0 | 0 | 0 | 0 | 0 |
| 6 | | 0 | 0 | 0 | 0 | 0 | 0 | 0 | 0 | 0 | 0 | 0 | 0 | 0 |
| 7 | | 0 | 0 | 0 | 0 | 0 | 0 | 0 | 0 | 0 | 0 | 0 | 0 | 0 |
| 8 | | 0 | 0 | 0 | 0 | 0 | 0 | 0 | 0 | 0 | 0 | 0 | 0 | 0 |
| 9 | | 0 | 0 | 0 | 0 | 0 | 0 | 0 | 0 | 0 | 0 | 0 | 0 | 0 |
| 10 | | 0 | 0 | 0 | 0 | 0 | 0 | 0 | 0 | 0 | 0 | 0 | 0 | 0 |
| 11 | | 0 | 0 | 0 | 0 | 0 | 0 | 0 | 0 | 0 | 0 | 0 | 0 | 0 |
| 12 | | 0 | 0 | 0 | 0 | 0 | 0 | 0 | 0 | 0 | 0 | 0 | 0 | 0 |
| 13 | | 0 | 0 | 0 | 0 | 0 | 0 | 0 | 0 | 0 | 0 | 0 | 0 | 0 |
| 14 | | 0 | 0 | 0 | 0 | 0 | 0 | 0 | 0 | 0 | 0 | 0 | 0 | 0 |
| 15 | | 0 | 0 | 0 | 0 | 0 | 0 | 0 | 0 | 0 | 0 | 0 | 0 | 0 |
| 16 | | 0 | 0 | 0 | 0 | 0 | 0 | 0 | 0 | 0 | 0 | 0 | 0 | 0 |
| 17 | | 0 | 0 | 0 | 0 | 0 | 0 | 0 | 0 | 0 | 0 | 0 | 0 | 0 |
| 18 | | 0 | 0 | 1 | 0 | 0 | 0 | 0 | 0 | 0 | 0 | 0 | 0 | 1 |
| 19 | | 0 | 0 | 0 | 0 | 0 | 0 | 0 | 0 | 0 | 0 | 0 | 0 | 0 |
| 20 | | 0 | 0 | 2 | 0 | 0 | 0 | 0 | 0 | 0 | 0 | 0 | 0 | 2 |
| 21 | | 0 | 0 | 4 | 0 | 0 | 0 | 0 | 0 | 0 | 0 | 0 | 0 | 4 |
| 22 | | 0 | 0 | 3 | 0 | 0 | 1 | 0 | 0 | 0 | 0 | 0 | 0 | 4 |
| 23 | | 0 | 1 | 7 | 0 | 0 | 0 | 0 | 0 | 0 | 0 | 1 | 0 | 9 |
| 24 | | 1 | 7 | 12 | 0 | 1 | 3 | 0 | 0 | 0 | 1 | 1 | 0 | 25 |
| 25 | | 0 | 3 | 11 | 0 | 0 | 1 | 0 | 0 | 1 | 0 | 0 | 1 | 17 |
| 26 | | 1 | 5 | 12 | 0 | 1 | 0 | 0 | 0 | 1 | 1 | 1 | 2 | 23 |
| 27 | | 1 | 10 | 28 | 0 | 2 | 3 | 0 | 0 | 1 | 2 | 0 | 1 | 47 |
| 28 | | 1 | 15 | 24 | 0 | 2 | 6 | 0 | 0 | 2 | 2 | 2 | 1 | 54 |
| 29 | | 6 | 38 | 54 | 0 | 8 | 5 | 0 | 1 | 7 | 0 | 3 | 4 | 120 |
| 30 | | 10 | 91 | 91 | 1 | 3 | 11 | 0 | 1 | 2 | 0 | 1 | 4 | 205 |
| 31 | | 8 | 57 | 49 | 1 | 5 | 6 | 0 | 0 | 1 | 0 | 3 | 1 | 123 |
| 32 | | 2 | 33 | 50 | 0 | 4 | 6 | 0 | 1 | 4 | 1 | 0 | 0 | 99 |
| 33 | | 6 | 19 | 52 | 0 | 6 | 8 | 0 | 2 | 1 | 0 | 4 | 0 | 92 |
| 34 | | 5 | 74 | 126 | 1 | 7 | 15 | 0 | 0 | 4 | 2 | 10 | 4 | 243 |
| 35 | | 15 | 129 | 130 | 0 | 6 | 14 | 0 | 0 | 7 | 2 | 5 | 4 | 297 |
| 36 | | 7 | 46 | 77 | 0 | 6 | 18 | 0 | 1 | 2 | 1 | 3 | 3 | 157 |
| 37 | | 2 | 27 | 76 | 1 | 8 | 17 | 0 | 1 | 3 | 1 | 5 | 0 | 139 |
| 38 | | 3 | 25 | 76 | 0 | 16 | 23 | 0 | 1 | 3 | 4 | 7 | 2 | 157 |
| 39 | | 6 | 62 | 129 | 1 | 6 | 20 | 0 | 4 | 4 | 3 | 9 | 1 | 239 |
| 40 | | 7 | 145 | 149 | 1 | 9 | 27 | 0 | 1 | 6 | 2 | 12 | 8 | 360 |
| 41 | | 9 | 42 | 85 | 1 | 8 | 20 | 0 | 2 | 6 | 2 | 3 | 2 | 171 |
| 42 | | 5 | 21 | 71 | 1 | 8 | 19 | 0 | 0 | 5 | 4 | 9 | 0 | 138 |
| 43 | | 2 | 22 | 64 | 0 | 9 | 22 | 0 | 1 | 7 | 2 | 10 | 2 | 139 |
| 44 | | 3 | 34 | 83 | 1 | 10 | 22 | 0 | 2 | 7 | 7 | 8 | 1 | 175 |
| 45 | | 14 | 95 | 138 | 0 | 10 | 23 | 0 | 4 | 12 | 3 | 6 | 4 | 295 |
| 46 | | 3 | 31 | 58 | 1 | 4 | 16 | 0 | 1 | 9 | 6 | 9 | 1 | 136 |
| 47 | | 4 | 12 | 29 | 0 | 8 | 16 | 0 | 0 | 7 | 6 | 7 | 0 | 85 |
| 48 | | 1 | 16 | 57 | 0 | 11 | 18 | 0 | 3 | 13 | 3 | 11 | 4 | 136 |
| 49 | | 0 | 17 | 57 | 1 | 5 | 27 | 0 | 4 | 12 | 7 | 8 | 3 | 141 |
| 50 | | 4 | 49 | 85 | 1 | 6 | 22 | 0 | 1 | 10 | 5 | 10 | 5 | 194 |
| 51 | | 0 | 21 | 44 | 3 | 6 | 27 | 0 | 1 | 12 | 4 | 11 | 2 | 131 |
| 52 | | 2 | 11 | 31 | 0 | 14 | 19 | 0 | 2 | 10 | 4 | 10 | 2 | 103 |
| 53 | | 2 | 5 | 38 | 1 | 12 | 36 | 2 | 0 | 6 | 9 | 8 | 2 | 119 |
| 54 | | 2 | 12 | 49 | 0 | 5 | 33 | 1 | 7 | 10 | 6 | 10 | 0 | 133 |
| 55 | | 4 | 35 | 53 | 2 | 8 | 29 | 0 | 2 | 12 | 7 | 17 | 1 | 166 |
| 56 | | 2 | 11 | 31 | 0 | 5 | 19 | 0 | 2 | 12 | 4 | 13 | 2 | 99 |
| 57 | | 2 | 8 | 32 | 2 | 5 | 10 | 0 | 2 | 5 | 2 | 11 | 0 | 77 |
| 58 | | 1 | 7 | 34 | 0 | 5 | 17 | 3 | 3 | 8 | 8 | 12 | 1 | 98 |
| 59 | | 1 | 14 | 31 | 2 | 12 | 15 | 0 | 3 | 15 | 6 | 12 | 1 | 111 |
| 60 | | 1 | 25 | 45 | 0 | 9 | 17 | 1 | 0 | 8 | 8 | 3 | 2 | 118 |
| 61 | | 0 | 7 | 22 | 1 | 5 | 14 | 0 | 1 | 10 | 2 | 11 | 3 | 76 |
| 62 | | 1 | 3 | 30 | 0 | 5 | 16 | 0 | 2 | 7 | 8 | 4 | 0 | 75 |
| 63 | | 1 | 6 | 18 | 0 | 5 | 8 | 0 | 1 | 6 | 3 | 14 | 2 | 63 |
| 64 | | 1 | 3 | 20 | 4 | 6 | 18 | 0 | 2 | 7 | 5 | 11 | 1 | 77 |
| 65 | | 2 | 4 | 24 | 1 | 1 | 17 | 0 | 1 | 8 | 5 | 12 | 1 | 74 |
| 66 | | 2 | 0 | 15 | 0 | 9 | 14 | 0 | 1 | 6 | 7 | 10 | 2 | 64 |
| 67 | | 1 | 3 | 20 | 0 | 3 | 17 | 0 | 3 | 6 | 5 | 9 | 2 | 68 |
| 68 | | 1 | 2 | 8 | 0 | 5 | 15 | 0 | 3 | 13 | 5 | 5 | 0 | 56 |
| 69 | | 0 | 4 | 25 | 1 | 11 | 17 | 0 | 4 | 12 | 4 | 8 | 0 | 86 |
| 70 | | 0 | 4 | 20 | 4 | 8 | 17 | 0 | 4 | 5 | 4 | 13 | 3 | 82 |
| 71 | | 1 | 3 | 25 | 1 | 14 | 21 | 0 | 2 | 7 | 6 | 14 | 6 | 99 |
| 72 | | 0 | 0 | 17 | 1 | 11 | 19 | 0 | 4 | 12 | 3 | 6 | 2 | 75 |
| 73 | | 2 | 5 | 22 | 1 | 12 | 22 | 1 | 4 | 7 | 7 | 9 | 2 | 92 |
| 74 | | 0 | 6 | 14 | 0 | 7 | 21 | 0 | 4 | 5 | 4 | 13 | 1 | 75 |
| 75 | | 1 | 8 | 24 | 1 | 9 | 22 | 1 | 4 | 9 | 9 | 13 | 1 | 101 |
| 76 | | 2 | 1 | 13 | 1 | 10 | 16 | 0 | 3 | 18 | 6 | 14 | 4 | 86 |
| 77 | | 1 | 3 | 16 | 0 | 11 | 21 | 2 | 8 | 8 | 4 | 8 | 2 | 83 |
| 78 | | 2 | 1 | 15 | 1 | 4 | 24 | 0 | 5 | 11 | 6 | 15 | 1 | 83 |
| 79 | | 2 | 3 | 14 | 1 | 7 | 19 | 0 | 4 | 10 | 4 | 11 | 6 | 79 |
| 80 | | 4 | 4 | 12 | 1 | 3 | 18 | 0 | 5 | 15 | 8 | 9 | 6 | 81 |
| 81 | | 2 | 5 | 17 | 3 | 7 | 7 | 3 | 7 | 5 | 3 | 11 | 3 | 71 |
| 82 | | 0 | 1 | 13 | 0 | 5 | 10 | 0 | 4 | 6 | 7 | 6 | 4 | 56 |
| 83 | | 0 | 2 | 12 | 0 | 6 | 11 | 1 | 5 | 11 | 5 | 7 | 1 | 61 |
| 84 | | 3 | 0 | 8 | 0 | 7 | 10 | 1 | 6 | 6 | 5 | 8 | 4 | 55 |
| 85 | | 0 | 0 | 14 | 0 | 6 | 7 | 0 | 1 | 7 | 5 | 7 | 2 | 49 |
| 86 | | 0 | 2 | 9 | 0 | 6 | 10 | 1 | 1 | 6 | 5 | 1 | 4 | 45 |
| 87 | | 0 | 1 | 3 | 1 | 1 | 6 | 0 | 2 | 6 | 6 | 4 | 6 | 36 |
| 88 | | 0 | 0 | 3 | 1 | 1 | 6 | 2 | 1 | 2 | 1 | 6 | 4 | 27 |
| 89 | | 0 | 1 | 3 | 1 | 2 | 3 | 0 | 0 | 3 | 3 | 3 | 1 | 20 |
| 90 | | 0 | 1 | 0 | 1 | 1 | 3 | 0 | 1 | 1 | 2 | 1 | 6 | 17 |
| 91 | | 0 | 0 | 6 | 1 | 2 | 0 | 1 | 2 | 3 | 1 | 1 | 4 | 21 |
| 92 | | 0 | 1 | 1 | 0 | 0 | 5 | 0 | 1 | 1 | 2 | 1 | 3 | 15 |
| 93 | | 0 | 0 | 2 | 1 | 1 | 0 | 0 | 1 | 2 | 0 | 2 | 0 | 9 |
| 94 | | 1 | 0 | 0 | 0 | 0 | 0 | 0 | 3 | 1 | 1 | 0 | 1 | 6 |
| 95+ | | 0 | 0 | 0 | 0 | 0 | 1 | 0 | 1 | 2 | 1 | 3 | 3 | 11 |

FIGO = International Federation of Gynecology and Obstetrics.
