# Supplementary material for: The health impact of human papillomavirus vaccination in the situation of primary human papillomavirus screening: A mathematical modeling study
Source: PLoS One. 2018 Sep 4;13(9):e0202924. doi: 10.1371/journal.pone.0202924 (PMC6122803; doi:10.1371/journal.pone.0202924)
Supplement: S3 Table — Age-specific test results and histological diagnoses of cervical cancer screening in the Netherlands in years 2000–2007, for (A) women’s first lifetime screen and (B) subsequent screens. Data were obtained from the nationwide registry of histo- and cytolopathology in the Netherlands (PALGA). (DOCX) [file pone.0202924.s008.docx]

**S3 Table. Age-specific test results and histological diagnoses of cervical cancer screening in the Netherlands in years 2000-2007, for (A) women’s first lifetime screen and (B) subsequent screens.** Data were obtained from the nationwide registry of histo- and cytolopathology in the Netherlands (PALGA).

A.

| **Cytology test result** | **Normal** | **ASCUS/**  **LSIL** | **HSIL** | **Normal** | **ASCUS/**  **LSIL** | **HSIL** | **Normal** | **ASCUS/**  **LSIL** | **HSIL** | **Normal** | **ASCUS/**  **LSIL** | **HSIL** | **Normal** | **ASCUS/**  **LSIL** | **HSIL** | **Normal** | **ASCUS/**  **LSIL** | **HSIL** | **Normal** | **ASCUS/**  **LSIL** | **HSIL** |
| --- | --- | --- | --- | --- | --- | --- | --- | --- | --- | --- | --- | --- | --- | --- | --- | --- | --- | --- | --- | --- | --- |
| Histological diagnosis | No histology | | | CIN 1 | | | CIN 2 | | | CIN 3 | | | Cervical cancer | | | Other cancer | | | CIN 0 (No CIN) | | |
| **Age group** |  |  |  |  |  |  |  |  |  |  |  |  |  |  |  |  |  |  |  |  |  |
| 24-28 | 5 | 0 | 0 | 0 | 0 | 0 | 0 | 0 | 0 | 0 | 0 | 0 | 0 | 0 | 0 | 0 | 0 | 0 | 0 | 1 | 0 |
| 29-33 | 33,354 | 848 | 53 | 0 | 128 | 33 | 0 | 97 | 92 | 0 | 101 | 333 | 0 | 1 | 13 | 0 | 0 | 0 | 0 | 69 | 13 |
| 34-38 | 8,001 | 193 | 17 | 0 | 20 | 8 | 0 | 13 | 15 | 0 | 16 | 82 | 0 | 1 | 5 | 0 | 0 | 0 | 0 | 13 | 4 |
| 39-43 | 3,464 | 109 | 13 | 0 | 7 | 3 | 0 | 3 | 4 | 0 | 5 | 33 | 0 | 1 | 3 | 0 | 0 | 1 | 0 | 11 | 1 |
| 44-48 | 2,394 | 60 | 8 | 0 | 4 | 1 | 0 | 2 | 6 | 0 | 6 | 12 | 0 | 1 | 5 | 0 | 0 | 0 | 0 | 5 | 3 |
| 49-53 | 1,482 | 37 | 3 | 0 | 1 | 2 | 0 | 1 | 2 | 0 | 1 | 5 | 0 | 1 | 3 | 0 | 0 | 0 | 0 | 1 | 0 |
| 54-58 | 1,031 | 21 | 2 | 0 | 1 | 0 | 0 | 0 | 0 | 0 | 0 | 4 | 0 | 1 | 1 | 0 | 0 | 0 | 0 | 4 | 1 |
| 59-63 | 897 | 11 | 2 | 0 | 0 | 0 | 0 | 1 | 0 | 0 | 1 | 5 | 0 | 0 | 1 | 0 | 0 | 0 | 0 | 1 | 0 |

ASCUS =Atypical squamous cells of undetermined significance; LSIL = low-grade squamous intraepithelial lesion; HSIL = high-grade squamous intraepithelial lesion

B.

| **Cytology test result** | **Normal** | **ASCUS/**  **LSIL** | **HSIL** | **Normal** | **ASCUS/**  **LSIL** | **HSIL** | **Normal** | **ASCUS/**  **LSIL** | **HSIL** | **Normal** | **ASCUS/**  **LSIL** | **HSIL** | **Normal** | **ASCUS/**  **LSIL** | **HSIL** | **Normal** | **ASCUS/**  **LSIL** | **HSIL** | **Normal** | **ASCUS/**  **LSIL** | **HSIL** |
| --- | --- | --- | --- | --- | --- | --- | --- | --- | --- | --- | --- | --- | --- | --- | --- | --- | --- | --- | --- | --- | --- |
| Histological diagnosis | No histology | | | CIN 1 | | | CIN 2 | | | CIN 3 | | | Cervical cancer | | | Other cancer | | | CIN 0 (No CIN) | | |
| **Age group** |  |  |  |  |  |  |  |  |  |  |  |  |  |  |  |  |  |  |  |  |  |
| 29-33 | 102,344 | 2,001 | 68 | 0 | 310 | 81 | 1 | 290 | 233 | 0 | 366 | 871 | 0 | 6 | 29 | 0 | 0 | 0 | 0 | 162 | 49 |
| 34-38 | 344,189 | 5,398 | 162 | 0 | 748 | 188 | 0 | 674 | 498 | 0 | 749 | 1,836 | 0 | 25 | 94 | 0 | 0 | 0 | 0 | 485 | 112 |
| 39-43 | 396,638 | 6,238 | 134 | 0 | 798 | 196 | 0 | 565 | 435 | 0 | 557 | 1,422 | 1 | 30 | 96 | 0 | 0 | 0 | 0 | 559 | 116 |
| 44-48 | 375,814 | 6,330 | 130 | 0 | 632 | 133 | 0 | 353 | 280 | 0 | 285 | 796 | 0 | 19 | 81 | 0 | 2 | 1 | 2 | 681 | 110 |
| 49-53 | 334,005 | 5,320 | 146 | 0 | 433 | 128 | 0 | 200 | 152 | 0 | 126 | 413 | 1 | 15 | 51 | 0 | 1 | 3 | 1 | 566 | 124 |
| 54-58 | 294,690 | 3,159 | 182 | 1 | 263 | 77 | 0 | 94 | 99 | 0 | 77 | 232 | 0 | 7 | 40 | 0 | 3 | 9 | 1 | 347 | 124 |
| 59-63 | 225,737 | 1,694 | 137 | 0 | 109 | 56 | 0 | 51 | 64 | 0 | 48 | 145 | 0 | 8 | 28 | 0 | 1 | 6 | 0 | 192 | 66 |

ASCUS =Atypical squamous cells of undetermined significance; LSIL = low-grade squamous intraepithelial lesion; HSIL = high-grade squamous intraepithelial lesion
