# Supplementary material for: The health impact of human papillomavirus vaccination in the situation of primary human papillomavirus screening: A mathematical modeling study
Source: PLoS One. 2018 Sep 4;13(9):e0202924. doi: 10.1371/journal.pone.0202924 (PMC6122803; doi:10.1371/journal.pone.0202924)
Supplement: S4 Table — (DOCX) [file pone.0202924.s009.docx]

**S4 Table. Transitions and duration distributions used in MISCAN-Cervix.**

| **Transition number^a^** | **Disease pathway^a^** | **From state** | **To state** | **Probability of transition** | **Type of distribution** | **Mean duration (years)** | **Weibull shape parameter** |
| --- | --- | --- | --- | --- | --- | --- | --- |
| 1 | A | CIN 0 HPV+ | Cleared/regressed | 1 | Exponential | 1 | 1 |
| 2 | B, C, D | CIN 0 HPV+ | Cleared/regressed | 1 | Exponential | 1 | 1 |
| 3 | B, C, D, F | CIN 0 HPV+ | CIN 1 HPV+ | 1 | Exponential | 1 | 1 |
| 4 | B | CIN 1 HPV+ | CIN 1 HPV- | 0.4 | Exponential | 1.5 | 1 |
| 5 | B | CIN 1 HPV+ | CIN 0 HPV+ | 0.3 | Exponential | 1.5 | 1 |
| 6 | B | CIN 1 HPV+ | Cleared/regressed | 0.3 | Exponential | 1.5 | 1 |
| 7 | B | CIN 1 HPV- | Cleared/regressed | 1 | Exponential | 1 | 1 |
| 8 | E | CIN 1 HPV- | Cleared/regressed | 1 | Exponential | 1.5 | 1 |
| 9 | C, D, F | CIN 1 HPV+ | CIN 2 HPV+ | 1 | Exponential | 1.5 | 1 |
| 10 | C | CIN 2 HPV+ | CIN 2 HPV- | 0.4 | Exponential | 2 | 1 |
| 11 | C | CIN 2 HPV+ | CIN 0 HPV+ | 0.3 | Exponential | 2 | 1 |
| 12 | C | CIN 2 HPV+ | Cleared/regressed | 0.3 | Exponential | 2 | 1 |
| 13 | C | CIN 2 HPV- | Cleared/regressed | 1 | Exponential | 1 | 1 |
| 14 | D, F | CIN 2 HPV+ | CIN 3 HPV+ | 1 | Exponential | 2 | 1 |
| 15 | D | CIN 3 HPV+ | CIN 3 HPV- | 0.4 | Weibull | 3.1 | 1.67 |
| 16 | D | CIN 3 HPV+ | CIN 0 HPV+ | 0.3 | Weibull | 3.1 | 1.67 |
| 17 | D | CIN 3 HPV+ | Cleared/regressed | 0.3 | Weibull | 3.1 | 1.67 |
| 18 | D | CIN 3 HPV- | Cleared/regressed | 1 | Exponential | 1 | 1 |
| 19 | E | CIN 1 HPV- | CIN 2 HPV- | 1 | Exponential | 1.5 | 1 |
| 20 | E | CIN 2 HPV- | Cleared/regressed | 1 | Exponential | 2 | 1 |
| 21 | F | CIN 3 HPV+ | Preclinical FIGO 1A | 1 | Weibull | 11.8 | 1.67 |
| 22 | F | Preclinical FIGO 1A | Preclinical FIGO 1B | 1 | Exponential | 3.2 | 1 |
| 23 | F | Preclinical FIGO 1B | Preclinical FIGO 2+ | Age-specific^b^ | Exponential | 0.5 | 1 |
| 24 | F | Preclinical FIGO 1B | Clinical FIGO 1B | Age-specific^b^ | Exponential | 0.5 | 1 |
| 25 | F | Preclinical FIGO 2+ | Clinical FIGO 2+ | 1 | Exponential | 1.3 | 1 |
| 26 | F | Clinical FIGO 1B | Cervical cancer death | Age-specific^c^ | Piecewise uniform | Age-specific^d^ | - |
| 27 | F | Clinical FIGO 2+ | Cervical cancer death | Age-specific^c^ | Piecewise uniform | Age-specific^d^ | - |

^a^ See S3 Fig.

^b^ Transition probability depends on age; see S3A Table.

^c^ Transition probability depends on age; see S3B Table.

^d^ See S3C Table for the duration distribution.
