# Supplementary material for: The health impact of human papillomavirus vaccination in the situation of primary human papillomavirus screening: A mathematical modeling study
Source: PLoS One. 2018 Sep 4;13(9):e0202924. doi: 10.1371/journal.pone.0202924 (PMC6122803; doi:10.1371/journal.pone.0202924)
Supplement: S5 Table — Percentages in the table are estimated in the model calibration. Linear interpolation is used to determine the probabilities at intermediate ages. (DOCX) [file pone.0202924.s010.docx]

**S5 Table. Age-specific probability that cervical cancer is detected in stages FIGO 1B and FIGO 2+, given that it is clinically detected.** Percentages in the table are estimated in the model calibration. Linear interpolation is used to determine the probabilities at intermediate ages.

| **Age** | **Clinical detection in stage:** | |
| --- | --- | --- |
|  | **FIGO 1B** | **FIGO 2+** |
| 0 | 25.4% | 74.6% |
| 25 | 25.4% | 74.6% |
| 40 | 35.0% | 65.0% |
| 55 | 61.4% | 38.6% |
| 70 | 75.4% | 24.6% |
| 100 | 75.4% | 24.6% |
