# Supplementary material for: The health impact of human papillomavirus vaccination in the situation of primary human papillomavirus screening: A mathematical modeling study
Source: PLoS One. 2018 Sep 4;13(9):e0202924. doi: 10.1371/journal.pone.0202924 (PMC6122803; doi:10.1371/journal.pone.0202924)
Supplement: S6 Table — Linear interpolation is used to determine the probabilities at intermediate ages. Source: observed age-specific and stage-specific survival for the periods 1989–2002 and 2003–2009, obtained from the Dutch Cancer Registry. (DOCX) [file pone.0202924.s011.docx]

**S6 Table. Model assumptions for the age-specific probability that clinical FIGO 1B and FIGO 2+ cervical cancer will lead to death from cervical cancer (i.e. 100% - probability of long-term survival), in the absence of other-cause mortality.** Linear interpolation is used to determine the probabilities at intermediate ages. Source: observed age-specific and stage-specific survival for the periods 1989-2002 and 2003-2009, obtained from the Dutch Cancer Registry.

| **Age** | **Clinical FIGO 1B** | **Clinical FIGO 2+** |
| --- | --- | --- |
| 0 | 9.7% | 45.5% |
| 30 | 9.7% | 45.5% |
| 45 | 10.8% | 51.1% |
| 60 | 22.9% | 55.4% |
| 80 | 34.5% | 68.7% |
| 100 | 34.5% | 68.7% |
