# Supplementary material for: The health impact of human papillomavirus vaccination in the situation of primary human papillomavirus screening: A mathematical modeling study
Source: PLoS One. 2018 Sep 4;13(9):e0202924. doi: 10.1371/journal.pone.0202924 (PMC6122803; doi:10.1371/journal.pone.0202924)
Supplement: S7 Table — The values in this table represent the percentages of cervical cancer deaths that occur within a given number of years after the moment of clinical diagnosis. It is assumed that no cervical cancer mortality occurs more than 10 years after clinical diagnosis. Source: observed age-specific and stage-specific survival for the periods 1989–2002 and 2003–2009, obtained from the Dutch Cancer Registry. (DOCX) [file pone.0202924.s012.docx]

**S7 Table. Model assumptions for the duration distribution of clinical FIGO 1B and FIGO 2+ cervical cancer, if the transition to death from cervical cancer occurs.** The values in this table represent the percentages of cervical cancer deaths that occur within a given number of years after the moment of clinical diagnosis. It is assumed that no cervical cancer mortality occurs more than 10 years after clinical diagnosis. Source: observed age-specific and stage-specific survival for the periods 1989-2002 and 2003-2009, obtained from the Dutch Cancer Registry.

| **Years after detection** | **Clinical FIGO 1B** | **Clinical FIGO 2+** |
| --- | --- | --- |
| 1 | 10.4% | 37.6% |
| 2 | 36.5% | 64.6% |
| 3 | 47.9% | 78.1% |
| 4 | 61.5% | 84.5% |
| 5 | 78.3% | 88.5% |
| 6 | 84.4% | 90.5% |
| 7 | 90.3% | 93.3% |
| 8 | 93.1% | 96.4% |
| 10 | 100% | 100% |
