# Supplementary material for: The health impact of human papillomavirus vaccination in the situation of primary human papillomavirus screening: A mathematical modeling study
Source: PLoS One. 2018 Sep 4;13(9):e0202924. doi: 10.1371/journal.pone.0202924 (PMC6122803; doi:10.1371/journal.pone.0202924)
Supplement: S8 Table — The observed proportions are based on the studies of Coupé et al., who reported age-specific HPV prevalence in women aged 18–65 years, and Guan et al. who determined the distribution of HPV types in CIN and cervical cancer (large meta-analysis of studies with different age ranges)[30, 31]. HPV = human papillomavirus; CIN = cervical intraepithelial neoplasia. (DOCX) [file pone.0202924.s013.docx]

**S8 Table. The observed and estimated proportions of HPV-16 and HPV-18 in HPV infections without cytological abnormalities, and in CIN 1, CIN 2, CIN 3, and invasive cervical cancer in the population prior to vaccination in MISCAN-Cervix.** The observed proportions are based on the studies of Coupé *et al.,* who reported age-specific HPV prevalence in women aged 18-65 years, and Guan *et al.* who determined the

distribution of HPV types in CIN and cervical cancer (large meta-analysis of studies with different age ranges)**.** HPV = human papillomavirus; CIN = cervical intraepithelial neoplasia.

|  |  | **HPV infections without cytological abnormalities** | **CIN 1** | **CIN 2** | **CIN 3** | **Cervical cancer** |
| --- | --- | --- | --- | --- | --- | --- |
| HPV-16 | Observed proportion | 25.4% | 15.4% | 37.6% | 47.2% | 62.5% |
|  | Estimated proportion | 25.5% | 16.5% | 28.1% | 46.5% | 62.7% |
|  |  |  |  |  |  |  |
| HPV-18 | Observed proportion | 8.2% | 7.8% | 7.4% | 4.7% | 17.2% |
|  | Estimated proportion | 8.3% | 9.3% | 6.9% | 7.0% | 17.5% |
