# Supplementary material for: The health impact of human papillomavirus vaccination in the situation of primary human papillomavirus screening: A mathematical modeling study
Source: PLoS One. 2018 Sep 4;13(9):e0202924. doi: 10.1371/journal.pone.0202924 (PMC6122803; doi:10.1371/journal.pone.0202924)
Supplement: S9 Table — In the model, we assumed that 10% of women never attend screening at the general practitioner, and that 90% are potential attenders[29]. Of the non-attending women, 3% opt-in to receive a self-sampling kit. *Attendance at age 65 is assumed to be equal to the observed attendance at age 60. (DOCX) [file pone.0202924.s014.docx]

**S9 Table. Attendance in the cervical cancer screening program and self-sampling kit in the base case and sensitivity analyses, based on the observed screening attendance in 2013(9).** In the model, we assumed that 10% of women never attend screening at the general practitioner, and that 90% are potential attenders(5). Of the non-attending women, 3% opt-in to receive a self-sampling kit.

| **Age (years)** | **Base case** | |  | **Higher attendance** | |  | **Lower attendance** | |
| --- | --- | --- | --- | --- | --- | --- | --- | --- |
|  | **Attendance office-based HPV test** | **Self-sampling HPV test** |  | **Attendance office-based HPV test** | **Self-sampling HPV test** |  | **Attendance office-based HPV test** | **Self-sampling HPV test** |
| 30 | 58.9% | 7.3% |  | 70.7% | 10.2% |  | 47.1% | 5.7% |
| 35 | 64.4% | 8.4% |  | 77.3% | 13.2% |  | 51.6% | 6.2% |
| 40 | 71.1% | 10.4% |  | 85.3% | 20.5% |  | 56.9% | 7.0% |
| 45 | 75.6% | 12.3% |  | 90.7% | 32.1% |  | 60.4% | 7.6% |
| 50 | 77.8% | 13.5% |  | 93.3% | 45.0% |  | 62.2% | 7.9% |
| 55 | 76.7% | 12.9% |  | 92.0% | 37.5% |  | 61.3% | 7.8% |
| 60 | 75.6% | 12.3% |  | 90.7% | 32.1% |  | 60.4% | 7.6% |
| 65* | 75.6% | 12.3% |  | 90.7% | 32.1% |  | 60.4% | 7.6% |

*Attendance at age 65 is assumed to be equal to the observed attendance at age 60.
